# Double-dose osimertinib combined with intrathecal injection of pemetrexed improves the efficacy of EGFR-mutant non-small cell lung cancer and leptomeningeal metastasis: case report and literature review

**DOI:** 10.3389/fonc.2024.1377451

**Published:** 2024-04-22

**Authors:** Wenjuan Zhong, Longqiu Wu, Lixing Huang, Jianfeng Wang, Huaqiu Shi, Shugui Wu

**Affiliations:** ^1^ Department of Oncology, The First Affiliated Hospital of Gannan Medical University, Ganzhou, Jiangxi, China; ^2^ Jiangxi Clinical Medical Center for Cancer, Ganzhou, Jiangxi, China; ^3^ Department of Gastroenterology, The First Affiliated Hospital of Gannan Medical University, Ganzhou, Jiangxi, China; ^4^ Department of Oncology, The Affiliated Ganzhou Hospital of Nanchang University, Ganzhou, Jiangxi, China

**Keywords:** non-small cell lung cancer, leptomeningeal metastasis, osimertinib, pemetrexed, intrathecal chemotherapy

## Abstract

Leptomeningeal metastasis (LM) is a complication of non-small cell lung cancer (NSCLC) characterized by poor prognosis and short survival. A variety of therapeutic approaches have been sought to improve the efficacy of LM. Here we present a clinical case and conduct a literature review to investigate the effectiveness and safety of double-dose osimertinib combined with a pemetrexed intrathecal injection. This is an older man who underwent thoracoscopic pneumonectomy and was diagnosed with stage IIA lung adenocarcinoma with EGFR21 L858R mutation. He experienced thoracic vertebral metastases 33 months postoperatively and received first-line treatment with gefitinib combined with radiotherapy for vertebral metastases. However, the patient developed a grade 3 rash with unacceptable toxicity and his CEA levels were significantly increased 22 months later, leading to a targeted treatment adjustment to 80 mg of osimertinib orally once daily. Four months later, the patient developed LM and osimertinib dosage was increased to 160 mg once daily; however, neurological symptoms did not improve, and cerebrospinal fluid (CSF) tumor cells remained detected. Accordingly, the patient received an intrathecal injection of pemetrexed (dose 30 mg) every 2-3 months, 2-3 times per course (4-6 days each time), and continued to receive a double dose of osimertinib. After three courses of intrathecal chemotherapy, CSF tumor cells were eliminated, and neurological symptoms significantly improved. During the treatment, he experienced a one-degree rash, leukopenia, thrombocytopenia, and fatigue. This patient has been alive and well with disease control for 28 months since the diagnosis of meningeal metastases. Combining double-dose osimertinib and an intrathecal injection of pemetrexed demonstrated therapeutic efficacy and manageable adverse effects in this patient with advanced NSCLC with EGFR-mutant and LM.

## 1 Introduction

Leptomeningeal metastasis (LM) is a rare and devastating complication of metastatic non-small cell lung cancer (NSCLC). The incidence of LM in patients is approximately 3-5%, and patients harboring epidermal growth factor receptor (EGFR) mutations and anaplastic lymphoma kinase (ALK) rearrangements are more likely to progress to LM (1–3). Owing to the lack of specific clinical manifestations, early diagnosis of LM remains difficult and challenging, often leading to misdiagnosis or missed diagnosis. In central nervous system metastases, the median overall survival (OS) is approximately 12 months in patients with brain metastases (4), whereas the median OS is only 3 months in patients with LM (2). Treatment options include radiotherapy, chemotherapy, intrathecal treatment, molecular targeted therapy, and immunotherapy. However, owing to the blood-brain barrier (BBB), most drugs face challenges in penetrating the meningeal cavity, resulting in limited treatment efficacy and a median survival of only a few months. Therefore, tyrosine kinase inhibitors (TKIs) with enhanced BBB permeability and more effective drugs are urgently needed to enhance the therapeutic efficacy against LM.

Osimertinib is a third-generation irreversible epidermal growth factor receptor tyrosine kinase inhibitor (EGFR-TKI) that effectively and selectively inhibits EGFR-TKI-sensitizing and EGFR T790M mutations. High doses of osimertinib exhibit high BBB penetration rates. The BLOOM phase I study demonstrated that a doubled dose of osimertinib (160 mg/day) exhibits significant efficacy in NSCLC patients with EGFR-mutant LM, with an objective response rate of 41%, a median progression-free survival (PFS) of 8.6 months and a median OS of 11 months (5). Although the incidence of adverse reactions has increased, osimertinib has shown significant therapeutic efficacy and manageable safety. Pemetrexed is a cell cycle-specific antimetabolite antineoplastic drug that inhibits folate metabolism and is a multitarget antifolate agent. It is considered the primary treatment option for advanced lung adenocarcinomas. However, the CSF penetration rate of pemetrexed is extremely low, accounting for less than 2% of plasma levels (6). Intrathecal chemotherapy (IC) is a viable approach for treating LM; its advantage is that it can directly penetrate the blood-CSF barrier and maximize drug exposure in the CSF. A direct intrathecal injection of pemetrexed has demonstrated effectiveness in the treatment of LM (7). Currently, there is limited literature available regarding the intrathecal injection of pemetrexed for the treatment of LM in NSCLC. Here we present a case report of lung adenocarcinoma with an EGFR mutation, who developed meningeal metastasis and received double-dose osimertinib combined with intrathecal injection of pemetrexed to enhance therapeutic efficacy.

## 2 Case presentation

**Figure 1:**
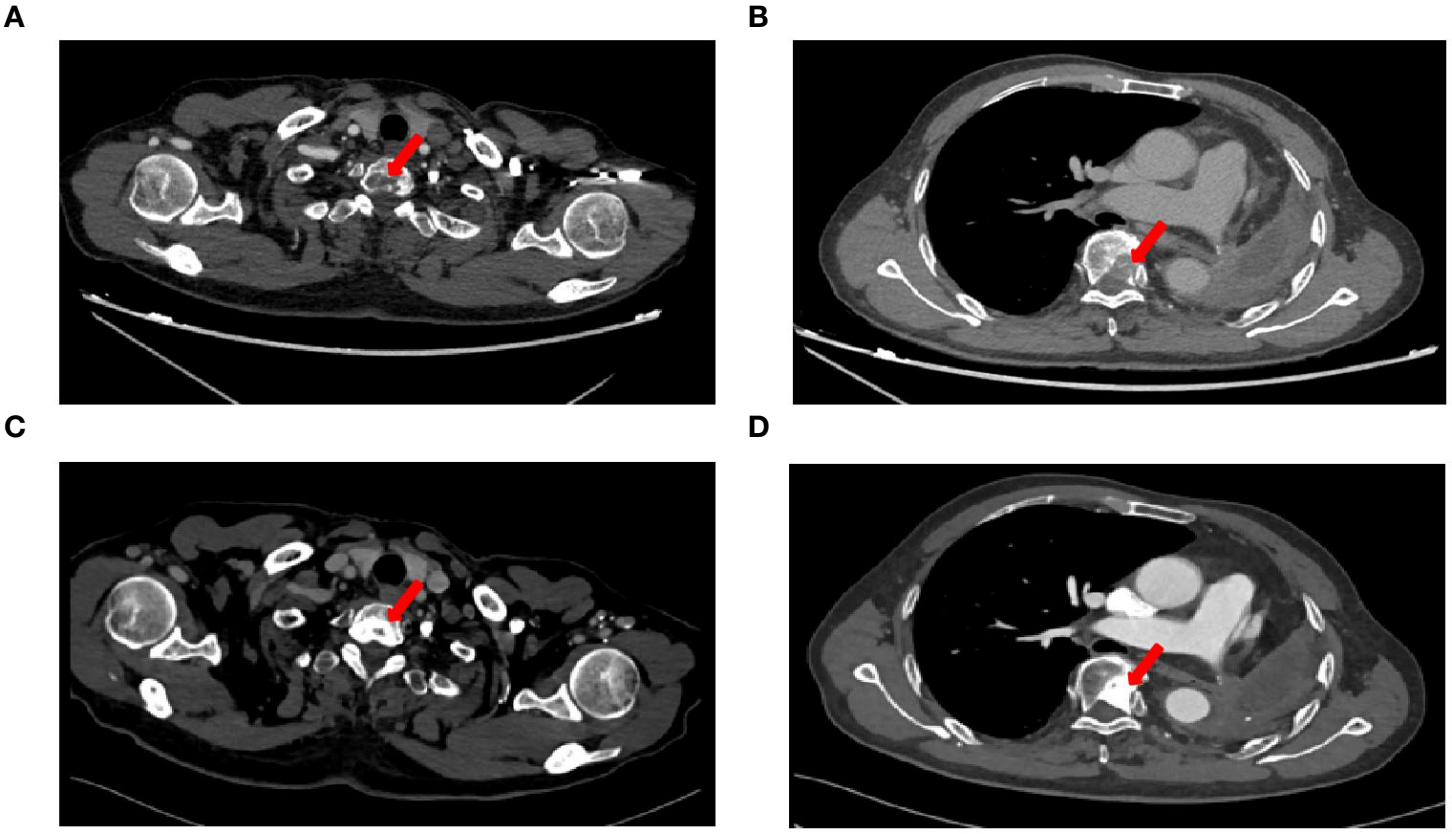
CT imaging changes before and after treatment. **(A)** Chest CT images on 23 July, 2019 showed T1 vertebral metastasis, **(B)** Chest CT images on 23 July, 2019 showed T6 vertebral metastasis, **(C)** Chest CT images showed osteogenic changes of T1 vertebral metastasis after radiotherapy, **(D)** Chest CT images showed osteogenic changes of T6 vertebral metastasis after radiotherapy.

**Figure 2:**
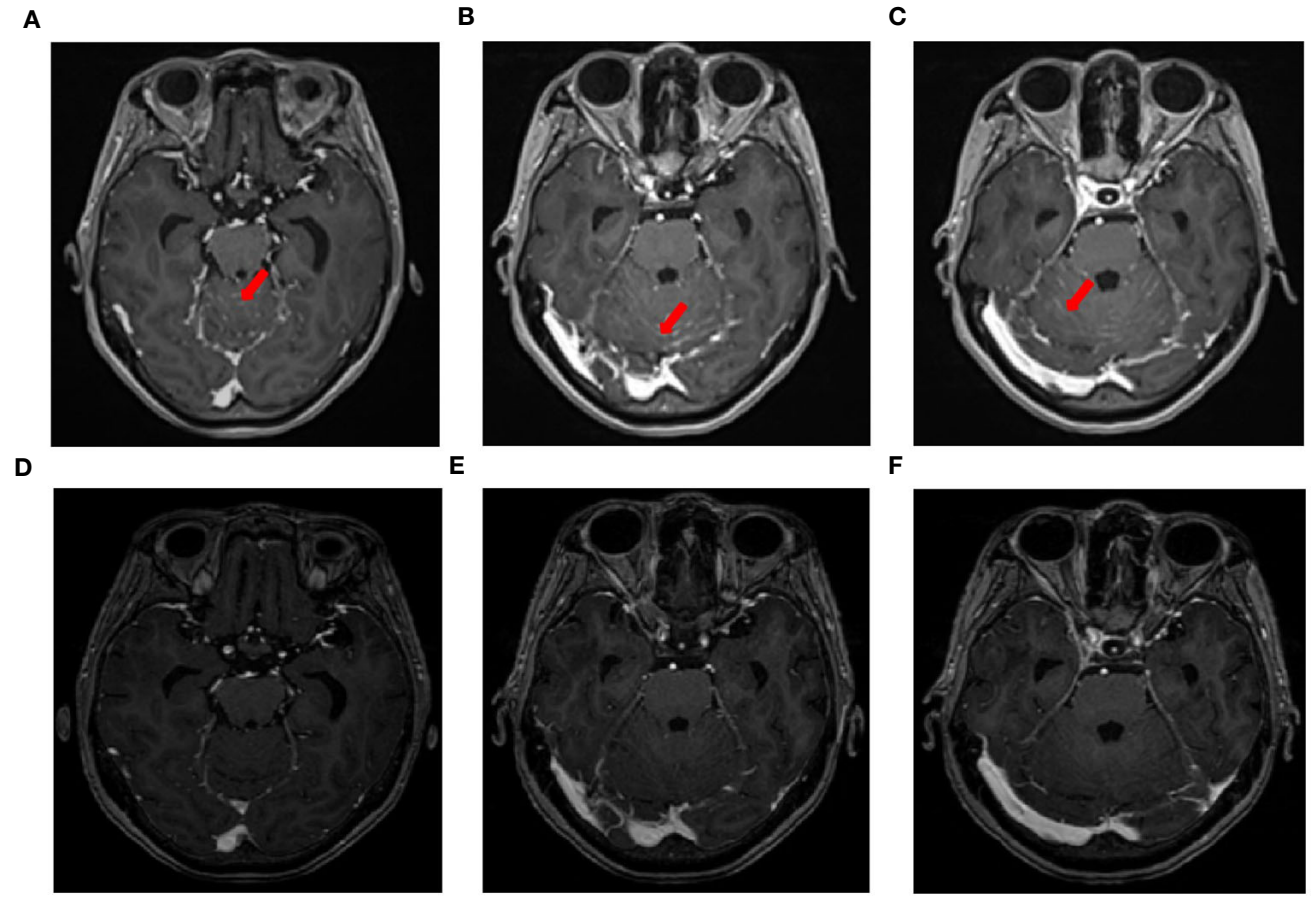
MRI changes in leptomeningeal metastasis before and after treatment. **(A–C)** MRI images of leptomeningeal metastasis in September 2021 showing significant thickening and pronounced enhancement of the meninges in the cerebellum. **(D–F)** After three courses of intrathecal chemotherapy, the thickening and pronounced enhancement of the meninges in the cerebellum disappeared.

**Figure 3:**
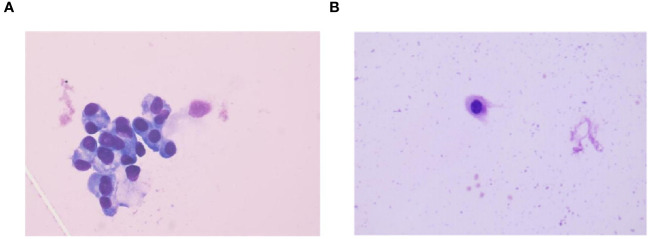
CSF cytology findings before and after treatment. **(A)** CSF cytology findings in September 2021 reveal tumor cells (HE staining,×100). **(B)** CSF cytology showing reactivity changes after three courses of intrathecal chemotherapy (HE staining,×100).

**Figure 4:**
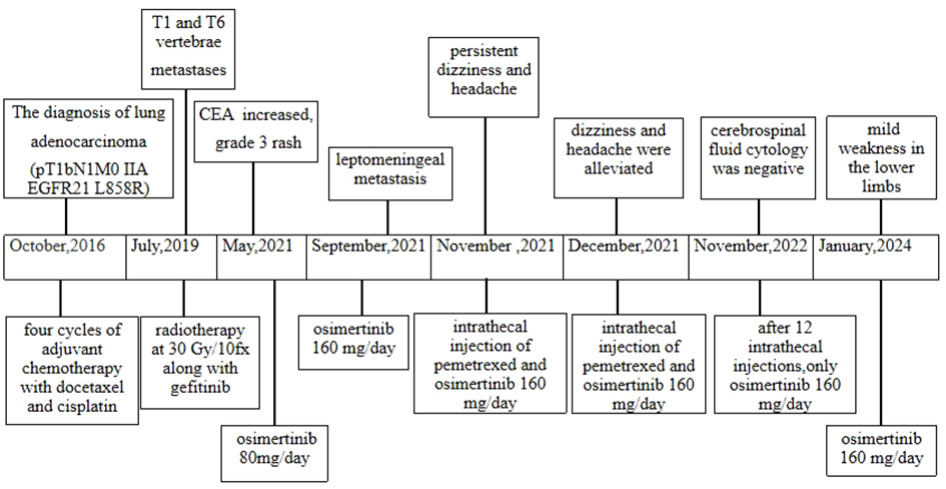
Timeline of the treatment process.

A 71-year-old male, with a smoking history of over 40 years, was admitted to The First Affiliated Hospital of Gannan Medical University (Ganzhou, China) on October 16, 2016, with a persistent cough lasting for 9 months and hemoptysis for 1 week. CT revealed a mass in the upper lobe of the left lung. On October 20, 2016, the patient underwent a total resection of the left lung and systemic lymph node dissection under general anesthesia. Postoperative pathology revealed lung adenocarcinoma, and one-third of the hilar lymph node metastases was positive. Immunohistochemistry revealed TTF-1 (+), CK7 (+), Napsin A (+), and Ki-67 expression (approximately 20%). The postoperative diagnosis was stage IIA (pT1bN1M0), according to the 7th edition of the TNM classification for lung cancer. Genetic testing using ARMS revealed the EGFR21 L858R mutation. After surgery, the patient received four cycles of adjuvant chemotherapy with docetaxel and cisplatin. On July 23, 2019, the patient presented with headache and pain in the neck and chest. MRI and CT scans indicated metastases in the T1 and T6 vertebrae (**Figures 1A, B**). Subsequently, the patient underwent radiotherapy for vertebral metastases at a dose of 30 Gy/10 fx along with molecular-targeted therapy using gefitinib (Iressa, 250 mg/day). During regular follow-up examinations, the disease remained stable, with osteogenic changes observed in the thoracic vertebral metastases (**Figures 1C, D**). In May 2021, CEA levels increased from 9.7 ng/ml to 214 ng/ml, accompanied by the onset of a grade 3 rash following the oral administration of gefitinib. Consequently, target therapy was switched to osimertinib at 80 mg/day. After one month, the CEA decreased to 161.5 ng/ml and the rash improved to grade 1. In September 2021, the patient presented with dizziness, headache, nausea, vomiting, and weakness in both lower extremities. Magnetic resonance imaging (MRI) revealed significant thickening and pronounced enhancement of the meninges in the cerebellum (**Figures 2A–C**). A lumbar puncture confirmed the presence of cancer cells in the cerebral effusion, suggesting meningeal metastasis (**Figure 3A**). Second-generation CSF sequencing (OncoDrug-Seq sequencing platform: Illumina NextSeq500vaSeq) identified an EGFR21 L858R mutation with an abundance of 38% in combination with TP53 and FGFR3 mutations. Subsequently, the patient received a double dose of osimertinib (160 mg/day) for 2 months; however, there was no significant improvement in the dizziness or headache. Starting in November 25, 2021, the patient received an intrathecal injection of 30 mg pemetrexed, administered 1-2 times per week, 2-3 times per course and repeated once every 2-3 months. Additionally, the patient was prescribed one centrum tablet daily and continued targeted therapy with osimertinib (160 mg/day). Following one course of intrathecal chemotherapy, the symptoms of dizziness and headache were alleviated, and after three courses of intrathecal chemotherapy, the thickening and pronounced enhancement of the meninges in the cerebellum disappeared (**Figures 2D–F**), and CSF cytology was negative (**Figure 3B**). The patient underwent a total of 12 intrathecal injections of pemetrexed from November 2021 to November 2022. The patient has a good quality of life, and no disease progression is observed. Adverse drug reactions include grade 1 rash, grade 1 leukopenia, grade 1 thrombocytopenia, and fatigue. Considering the absence of evident neurological symptoms and negative CSF cytology, intrathecal pemetrexed chemotherapy is discontinued, and only 160 mg osimertinib targeted therapy is currently being utilized. The patient exhibites mild weakness of the lower limbs as the main clinical symptom with no apparent adverse drug reactions. Since the diagnosis of LM, the patient has survived for 28 months (**Figure 4**).

## 3 Discussion

Patients with LM are primarily diagnosed based on clinical symptoms, MRI of the brain and spine, and CSF analysis. Typical cranial MRI enhancements include leptomeningeal, subependymal, dural, or cranial nerve enhancement; superficial cerebral lesions; and communicating hydrocephalus (8). The sensitivity and specificity of MRI for detecting LM in solid tumors are 70–87% and 75–94%, respectively (9, 10). In this patient, MRI revealed significant thickening and pronounced enhancement of the meninges in the cerebellum. The gold standard for LM diagnosis is the detection of tumor cells in the CSF. Initially, the CSF yielded a positive rate of approximately 50% upon the first examination, which increased to 80% with two consecutive tests. However, conducting more than three examinations within a short period does not increase the positivity rate (11). In addition, rapid *in vitro* fixation of lumbar puncture samples is crucial for enhancing the accuracy of the cytopathological diagnosis of CSF. An increasing number of molecular assays have been developed to enhance detection specificity and sensitivity. CSF analysis of epithelial cell adhesion molecules (EpCAMs), circulating tumor DNA (ctDNA), vascular endothelial growth factor **(VEGF)**, and C3 mRNA can significantly improve the sensitivity and detection rate of LM (12–16).

Currently, no standard treatment options are available for lung cancer patients with LM. Common treatment options include radiotherapy, targeted therapy, immunotherapy, and chemotherapy, either as a monotherapy or in combination. However, the efficacy of these treatments is not ideal, resulting in a median survival time ranging from 3 to 11 months (2, 17). Radiotherapy is considered a pivotal therapy for LM, as it effectively diminishes the size of the LM, enhances CSF circulation, alleviates hydrocephalus, mitigates neurological symptoms, and ultimately ameliorates patients’ quality of life. Local radiotherapy is commonly employed to treat visible meningeal nodules, cranial nerve involvement at the skull base, and cauda equina syndrome. Whole brain radiotherapy (WBRT) is the primary treatment for patients with nodular leptomeningeal disease metastasis. However, the efficacy of WBRT remains unclear. Clinical studies have shown that the OS of patients who undergo WBRT and those who do not are 10.9 months and 2.4 months, respectively *(P=0.002)*, which significantly prolonged the OS of patients (18). However, other studies have shown that WBRT can control craniocerebral symptoms but does not improve OS in patients with NSCLC-LM (19–21). As WBRT can lead to higher neurocognitive dysfunction, stereotactic radiosurgery or large-segmenting radiotherapy is currently preferred. WBRT can be considered in patients with neurological symptoms who are in good physical condition and ineligible for local stereotactic radiosurgery or large-segmenting radiotherapy (22). The general clinical consensus on the radiation dose and fractionation of WBRT is 30 Gy/10 fx and 40 Gy/20 fx. According to the guidelines of the National Comprehensive Cancer Network (NCCN), 37.5 Gy/15 fx was adopted as the fractionation method (23).

Targeted drugs have significantly improved the OS of patients with NSCLC harboring LM and driver gene mutations. The ability of small-molecule TKIs to penetrate the BBB is greater than that of large-molecule chemotherapy drugs. However, it should be noted that many TKIs are substrates of P-glycoprotein, which serves as a protein pump responsible for drug efflux at the BBB. Consequently, most TKIs still encounter limitations in traversing the BBB owing to their interaction with P-glycoproteins. Drugs, such as gefitinib, erlotinib, afatinib, and crizotinib, have low BBB penetration; therefore, the pia is a frequent site of disease progression in many TKIs. Studies have revealed that during treatment, a significant proportion of patients (up to 45%) receiving EGFR-TKI therapy develop central nervous system (CNS) metastases, encompassing both brain metastases and LM (4, 24). Osimertinib demonstrated superior CNS efficacy compared with platinum-pemetrexed in T790M-positive advanced NSCLC (25). A retrospective analysis of studies across the AURA program reported that the objective LM response rate to osimertinib (80 mg/day) was **55%**. Median LM PFS and OS were 11.1 months and 18.8 months, respectively (26). In an EGFR mutant, PC9 mouse brain metastasis model, osimertinib demonstrated greater penetration than other first- or second-generation TKIs (27). Therefore, osimertinib is the preferred treatment for NSCLC patients with EGFR mutations with or without the T790M mutation. In the ALEX study, alectinib exhibited a median PFS of 25.4 months for patients with baseline CNS metastasis, compared to 7.4 months in the crizotinib group, resulting in a significant 40% reduction in the risk of mortality (28).

Immune checkpoint inhibitors (ICIs) have changed outcomes for locally advanced or metastatic NSCLC patients with negative driver genes by improving PFS, OS, and quality of life. Traditionally, the brain has been regarded as an immune-privileged organ; however, emerging evidence suggests that ICIs can penetrate the BBB when brain tumors or metastases disrupt their integrity, leading to alterations in the local tumor microenvironment (29). ICIs themselves do not directly target cancer cells; instead, they enhance the immune activity of T cells, facilitating their infiltration into the brain and pial metastasis tumors (30, 31), thereby exerting an immunotherapeutic effect. Owing to the exclusion of patients with LM in most clinical studies, limited data are available on the treatment of LM with ICIs. The brain response rate to Nivolumab in programmed death-1 (PD-1) positive NSCLC patients has been reported to be 33% (32). A retrospective analysis was conducted on a cohort of 1,288 patients with prior immunotherapy, among whom 19 (1.5%) had NSCLC-LM and received immunotherapy. The PFS rates at 6 and 12 months were 21.0% and 0%, respectively, and the OS rates were 36.8% and 21.1%, respectively (33). Combined immunotherapies have yielded promising results. A Phase II study investigated the efficacy of atezolizumab in combination with carboplatin and pemetrexed as first-line treatment for patients with initial brain metastases. This study included 40 patients with a median follow-up period of 30 months. The median intracranial PFS and median OS were 6.9 months and 11.8 months, respectively. The response rate was 42.7%, and the 2-year OS rate was 27.5% (34). Furthermore, clinical studies have supported the combination of immunotherapy and radiotherapy, demonstrating synergistic antitumor activities (35, 36). An initial Phase I trial (KEYNOTE-001) revealed that among 97 patients with advanced NSCLC who received pembrolizumab after radiotherapy, longer PFS and OS rates were observed, along with an acceptable safety profile (37).

Systemic chemotherapy is the primary treatment for driver gene mutation-negative NSCLC patients with meningeal metastasis. Pemetrexed, an anti-metabolic type of cancer drug, exerts its mechanism of action by inhibiting the synthesis of purines and pyrimidines through the blockade of three essential enzymes: thymidylate synthetase (TS), dihydrofolate reductase (DHFR), and glycinamide ribronucleotide formyltransferase(GARFT). This inhibition leads to cell cycle arrest in the S phase, effectively suppressing tumor cell growth (38)**. Pemetrexed demonstrates potent antitumor activity against non-squamous NSCLC.** A retrospective study revealed that the median OS was 13.7 months for patients treated with pemetrexed after LM, compared to 4.0 months for those not treated with pemetrexed. Furthermore, multivariate analysis demonstrated that pemetrexed use after LM was independently associated with longer survival (39). However, because of the presence of the BBB, most chemotherapeutic drugs face challenges in penetrating the leptomeningeal cavity, thereby limiting their therapeutic efficacy. The key to treating LM is penetrating the BBB and reaching an effective concentration in the CSF. The intrathecal injection of chemotherapeutic agents avoids the “hurdle” posed by the BBB, directly delivers the drug to the subarachnoid space, and is considered the most direct treatment. Intrathecally injected drugs include cytarabine, methotrexate, and thiotepa; however, their therapeutic effects remain unsatisfactory. Professor Pan (40) was the first to carry out a phase I clinical study of intrathecal chemotherapy with pemetrexed as a salvage therapy for NSCLC-LM. The study results demonstrated that the clinical response rate to 10 mg pemetrexed was 31% (4/13), and the disease control rate was 54% (7/13). Moreover, this treatment exhibited excellent tolerability and offers a novel and efficacious therapeutic approach for patients with NSCLC-LM. The intravaginal administration of pemetrexel enables the drug to bypass the BBB and directly enter the CSF, achieving effective concentrations with minimal systemic toxicity. Similarly, Professor Xin Tao’s team (7) also conducted a phase I/II clinical trial to evaluate the efficacy of intrathecal chemotherapy with pemetrexed for LM. In the Phase I study, six patients were enrolled, and the dose of intrathecal pemetrexed was escalated from 15 mg to 80 mg, ultimately recommending a dose of 50 mg. Thirty patients diagnosed with EGFR mutation-positive LM-NSCLC were enrolled in the Phase II study, and 26 patients were included in the efficacy evaluation. Each patient received 2–12 intrathecal injections, resulting in a median OS of 9.0 months. The clinical response rate was 84.6% (22/26), with 2 patients achieving a complete response and 7 achieving a partial response. Most adverse events observed in all patients were mild and predominantly myelo-suppressed. This trial demonstrated the efficacy of intrathecal pemetrexel injections in patients with EGFR-mutant NSCLC-LM.

The management of LM should be personalized, based on clinical manifestations, imaging, and CSF analysis, to guide the systemic treatment plan in combination with radiotherapy, immunotherapy, targeted therapy, or intrathecal therapy. In this case, after the occurrence of meningeal metastases, monotherapy with osimertinib at a dosage of 160 mg did not yield significant improvements in the symptoms of dizziness and headache; however, when combined with intrathecal injection of pemetrexel, cranial symptoms were effectively controlled while exhibiting controllable toxicity. Given the patient’s advanced age, pemetrexed was intrathecally administered at a dose of 30 mg. The BLOOM study indicates that osimertinib 160 mg was generally well tolerated, and the adverse drug reactions included rash, acne, diarrhea, nausea, and paronychia and were predominantly grade 1-2 (5). Osimertinib (160 mg/day) combined with intrathecal injection of pemetrexed chemotherapy did not significantly increase the adverse drug events, and the adverse drug reactions mainly were grade 1 rash, grade 1 leukopenia, grade 1 thrombocytopenia, and fatigue. Since the diagnosis of LM, the patient has undergone 12 intrathecal injections and has survived for 28 months, experiencing a significantly improved quality of life. We will continue to follow-up the patient. Intrathecal injection of pemetrexed may be a promising treatment strategy for patients with NSCLC-LM.

## 4 Conclusion

The combination of double-dose osimertinib and an intrathecal injection of pemetrexed demonstrated meaningful therapeutic efficacy and manageable adverse effects in this advanced NSCLC patient with EGFR-mutant and LM. However, its efficacy and safety require further validation and more clinical data.

## Data availability statement

The datasets presented in this study can be found in online repositories. The names of the repository/repositories and accession number(s) can be found in the article/**Supplementary Material**.

## Ethics statement

The studies involving humans were approved by Ethics Committee of the First Affiliated Hospital of Gannan Medical University. The studies were conducted in accordance with the local legislation and institutional requirements. The participants provided their written informed consent to participate in this study. Written informed consent was obtained from the individual(s) for the publication of any potentially identifiable images or data included in this article.

## Author contributions

WZ: Conceptualization, Data curation, Writing – original draft, Writing – review & editing. LW: Methodology, Supervision, Writing – review & editing. LH: Formal Analysis, Writing – review & editing, Investigation. JW: Software, Writing – review & editing. HS: Supervision, Validation, Writing – review & editing. SW: Conceptualization, Methodology, Validation, Writing – review & editing.

## Supplementary material

Supplementary Material 1 The trend of changes in CEA(ng/ml) in serum (normal levels, <5 ng/ml).
