# Supplementary figures and images for: Double-dose osimertinib combined with intrathecal injection of pemetrexed improves the efficacy of EGFR-mutant non-small cell lung cancer and leptomeningeal metastasis: case report and literature review
Source: Front Oncol. 2024 Apr 22;14:1377451. doi: 10.3389/fonc.2024.1377451 (PMC11070505; doi:10.3389/fonc.2024.1377451)

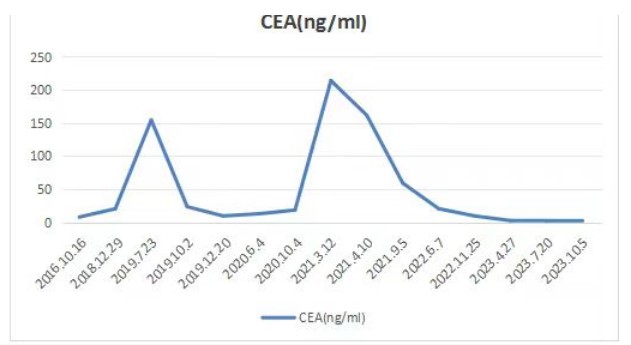

Supplement: Supplementary Material 1 — The trend of changes in CEA(ng/ml) in serum (normal levels, <5 ng/ml). [file Image_1.jpeg]
